# Fertility in Gyr Cows (*Bos indicus*) with Fixed Time Artificial Insemination and Visual Estrus Detection Using a Classification Table

**DOI:** 10.1155/2014/404363

**Published:** 2014-08-26

**Authors:** Lilido Nelson Ramírez-Iglesia, Rafael María Roman Bravo, Adelina Díaz de Ramirez, Leandro J. Torres

**Affiliations:** ^1^Centro de Investigaciones Agrícolas, Biológicas, Educativas y Sociales (CIABES), Universidad de Los Andes-Trujillo (ULA), Avenida Medina Angarita, Casa Carmona. Apartado Postal 198, Trujillo 3150, Estado Trujillo, Venezuela; ^2^Facultad de Ciencias Veterinarias, Núcleo Agropecuario, La Universidad del Zulia, Ciudad Universitaria, Avenida Goajira, Maracaibo 4001, Estado Zulia, Venezuela

## Abstract

The aim of this research was to compare two artificial insemination protocols (AIP): hormonal synchronization with fixed time artificial insemination (SC-FTAI) and the use of a table based on visual observation of estrus signs (VO) in order to identify cows in natural or spontaneous estrus being assigned to AI (NSE-IA). Two groups were formed: in the first group 109 cows were assigned to SC-FTAI, in which a commercial protocol is used; the second one included 108 randomly chosen cows, which were assigned to NSE-AI and in this group a modified table was used. Response variable was first service fertility rate (FSF), which was coded 1 for pregnant and 0 for empty. Predictor variables were AIP, postpartum anestrus, daily milk yield, body condition score at AI and calving number. Statistical analyses included association chi-square tests and logistic regression. Results showed an overall 41.94% FSF and a significant association was detected (*P* < 0.05) between FSF and daily milk yield; pregnancy rates were 42.20% and 41.67% for the SC-FTAI and NSE-IA groups, respectively (*P* > 0.05). The odds ratio for the effect of AIP was only 1.050, suggesting no differences in FSF between groups. The NSE-AI protocol can enhance both the technique of VO and reproductive efficiency. Further validation of the table is required.

## 1. Introduction

The Gyr (*Bos indicus*) cattle breed from India has been used pure or in crossbreeding programs with dairy breeds such as Holstein and Brown Swiss, in order to combine milk yield ability and adaptation genes that cannot be found in each breed alone. This practice has been helpful in the process to form that is known as dual purpose cattle (DP). In Venezuela, the dairy cattle of Gyr have been imported from Brazil. In DP farms, using artificial insemination (AI), estrus visual observation (VO) is routinely performed during the milking hours. The traditional and more widespread technique for detecting cows in estrus is the quiet acceptance (standing) to be mounted (QAM) by a bull or a herdmate and it is the only sign used for identifying the cows in estrus to be assigned to an AI program or to control natural mating [1–3]. It is the standard practice that cows that are not seen with QAM from a teaser bull or any herdmate are not considered in estrus and, therefore, are not served. This practice discards numerous secondary estrus signs which can be used to identify such a physiological state [2].

Despite the great progress that has been achieved in recent decades in understanding the reproductive physiology of the cow and its relationship to sexual behavior and ovulation [4] and fertility [5], along with a number of developed techniques for identification of cows in estrus [6, 7], inefficient heat detection continues to be present and it is a serious problem in reproductive management in DP farms, which is the main cause of reproductive failure under tropical environments [8] and the main cause of limiting fertility in the world [9, 10].

In zebu cattle there have been reported estrus, with weak signals, of short duration, and of low intensity; which is attributed to a low circulating levels of 17*β* estradiol [11, 12], which complicates estrous detection on cows [11]. Due to estrus detection problems, the solution has resorted to the use of hormonal treatments with fixed AI time (FTAI) without detecting estrus [13]; or the use of tables for rating estrus signs [14–17].

In the study of these new trends in reproductive technology of cattle, this investigation was set using a commercial Gyr herd with the objective of comparing first service fertility (FSF) and contrasting two artificial insemination protocols (AIP): (a) by applying an intravaginal commercial hormonal synchronization device and using FTAI and (b) by applying a table for rating the identified cows in estrus considering VO in addition to other secondary estrus signs. Thus, the effects on FSF, as days on postpartum anestrus, milk yield, body condition score at AI time, and parturition number, were investigated.

## 2. Materials and Methods

### 2.1. Data Source and Experimental Setting

In a Gyr (*Bos indicus*) cattle farm, located in a dry tropical forest area in the eastern basin of the Maracaibo lake, in the Bolivarian Republic of Venezuela, at 9°46′NL and 71°1′WL; at 0 m.a.s.l.; with bimodal annual rainfall pattern; annual temperature average of 28.5°C; relative humidity of 76 % and annual rainfall of 1,300 mm [18]. The herd consisted of Gyr milking line. Cows on study at the time of AI had an average ± standard deviation as follows: age, 7.7 ± 2.2 years; milk yield (DMY), 6.9 ± 2.1 kg/d; days of postpartum anestrus (PPA) 189 ± 83 days; calving number (CN), 3.6 ± 1.8 parturitions; and body condition at insemination (BCAI) of 2.5 ± 0.3 points, on a modified scale 0 = emaciated to 5 = obese, according to the adipose tissue in the pelvic area [19].

By VO during a period of 136 consecutive days, from December 2009 to March 2010, 108 cows in PPA, belonging to the same parlor, were inseminated after being detected in natural or spontaneous estrus (NSE-AI), according to the scoring criteria published previously [20, 21] Table 1.

**Figure 1:**
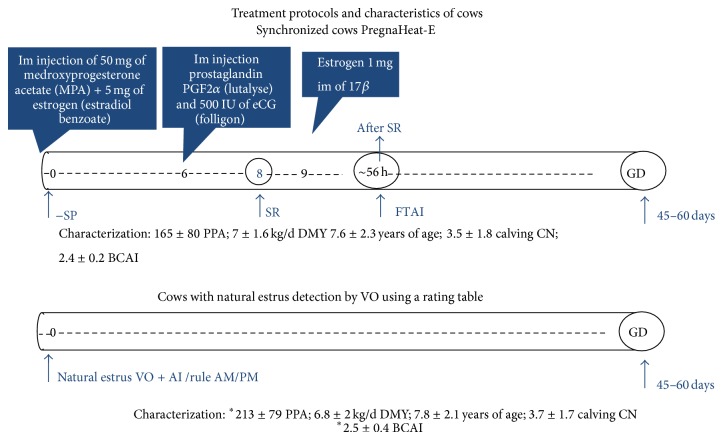
Treatment protocols and characteristics of cow groups. SP—sponge placed, SR—sponge removal, After SR—after sponge removal, FTAI—fixed time artificial insemination, AI—artificial insemination, PPA—postpartum anestrus, CN—calving number, BCIA—body condition at AI time, and GD—gestation diagnosis. ∗—significant differences (*P* < 0.05), Lutalyse and Folligon (pregnant mare serum gonadotropin (eCG)) and VO—visual observation.

During the same period, 109 cows in PPA were synchronized and inseminated at fixed time (SC-FTAI), in groups of ten cows at time, approximately every fifteen days, by using a (PH-E, PregnaHeat-E, VIATECA, Villa del Rosario, Venezuela) commercial intravaginal device, with protocol described by [21], which is contraindicated in cows with abnormalities or genital tract infections, less than 60 dpp and poor body condition (<2.5) Figure 1.

Cows were milked by hand twice a day with calf support, they were fed in paddocks sown rotation grasses* Brachiaria spp*, supplemented in holding pens for milking with a commercial concentrate for dairy cows (Procria)* ad libitum*, (minimum values of protein: 18–20%, fat: 4.6 %, NFE: 55 % and fiber: 5% max ). Minerals were supplied in canoes,* ad libitum*, as well as fresh water. In order to detect estrus signs, for purposes of applying Table 1, cows were observed daily in four periods: during the milking hours held from 6:00 to 09:00 am and from 3:00 to 06: 00 pm, and, at the paddock level, in the morning from 9:00 to 12:00 m and from 1:00 to 2:30 pm. A cow was considered in estrus if it scored at least 100 points; therefore, it was set aside for AI. The AI was performed according to the AM/PM rule. Semen from Gyr bulls was provided by traditional suppliers of the farm and the AI was conducted by experienced staff of the company. Pregnancy diagnosis was by transrectal palpation 45–60 days after AI. The observational period lasted until the diagnosis of pregnancy was made.

### 2.2. Statistical Analysis

Data were analyzed with the statistical analysis system (SAS) [22], by using the MEANS procedure, in order to perform Student's *t*-tests to describe and compare the characteristics of cows from the SC-FTAI and NSE-AI protocols. First service fertility (FSF) was coded either as empty (0) or as pregnant (1); predictor variables were classified into two levels as follows: postpartum anestrus (PPA: ≤100 and >100 days), daily milk yield (DMY: at AI; ≥6 and <6 kg/l/d), body condition score at AI (BCAI: ≥2.5 and <2.5), and calving number (CN: ≤3 and >3) and all these variables were coded as either 0 or 1, for each of their levels. In order to establish the degree of association between FSF and other classificatory variables, the SAS FREQ procedure was used, performing chi-square (*χ* ^2^) tests.

Binary classification of variables was based on the paradigmatic biological ideas in this part of the warm tropical climate, that considers as optimal: to have a calf per year; pregnancy achieved with PPA ≤ 100 days; a DMY > 6 kg/day; considering optimal a BCAI ≥ 2.5 for cows with CN ≤ 3.

In order to study the effects of binomial predictor variables: AIP, PPA, DMY, BCAI, and CN, on FSF, a logistic regression analysis was performed by using the SAS logistic procedure [22], in which the event of interest was 1 (pregnant). The estimate values of the odds ratio (OR) were used to compare the pregnancy odds between the levels of the effects. In practice, an OR = 1 is the absence of association or efficacy and values < 1 and > 1 as a low contribution and greater contribution for the event pregnant, respectively; 95% confidence intervals (95% CI) to indicate the limits between which is the value of the OR were also estimated. The data were processed at the Computer Centre of the University of the Andes Merida (CeCalCULA).

## 3. Results and Discussion 

Overall FSF for the herd was 41.94% (91/217). Moreover, it was 42.20% (46/109) for the SC-FTAI group and 41.67% (45/108) for the NSE-AI group, without significant differences between the two AIP from both *χ* ^2^ test and logistic regression (*P* > 0.05) (Table 2). In this research, the only effect significant on FSF was DMY (*P* < 0.05) and no association was found between FSF with PPA, BCIA, and CN (*P* > 0.05). However, results from *χ* ^2^ test may be seen cautiously, because they came from combining two way classification tables and this may be biased for other factors included that are under research.

A better inference may be found from the logistic regression analysis, because each factor is adjusted for the other factors included in the model. From the SAS logistic procedure, FSF probabilities can be modeled with the equation given in Table 3 and by straightforward manipulation with the appropriated values of the predictors, the different odds ratios shown there can be found.

Results for AIP may be seen appealing under the actual conditions of the country, because the first protocol may be more expensive than the other, which only demand trained personnel on the behavior of the cow during estrus. The odds ratio for the AIP was 1.050, (Table 3), which implies that there were no differences between the SC-FTAI when compared to the NSE-AI technique (*P* > 0.05), with only a superiority of 0.53% of the first group as compared to the other. The practical meaning of this finding is that either method should lead to the same results. Cows under the SC-FTAI protocol had a better nutritional status because cows chosen for that group had BCAI ≥ 2.5 BCAI (80%); this requirement was not considered in the NSE-AI protocol in which cows were only required to have at least 100 points imposed by the rating chart to be inseminated. By using the NSE-AI protocol it is probable to choose animals with optimal endocrine conditions for reproduction, because other signs of the sexual behavior of the cow are considered, in addition to the traditional practice of considering the QAM from a teaser bull or a herdmate as the only sign to be used. These reflections require more controlled studies to validate the weights assigned to those rating signs and to associate them to estrus with ovulation and/or pregnancy, as has been previously reported by [11, 14] for the ovulation time.

Higher pregnancy rates were found by using the same AI protocol, but without differences with the control treatment inseminated after VO of natural estrus [23–25]. On the other hand, [26] working with zebu synchronized animals and FTAI at 48 and 72 hours, they classified estrus intensity according to the number of mounts as high (≥20) and low (<20) without estrus by continuous VO; they detected 68.4% of cows in estrus; the overall pregnancy rate was only 25.4%; communicating these authors that 63.8% of the cows that did not show estrus, in fact ovulated, the functional biological phenomenon known as both silent estrus and ovulation occurred; on the other hand, 85.9% of cows with detected estrus ovulated, resulting in a 14% of cows showing the phenomenon known as an-ovulatory estrus which also has been reported for crossbred cows [27]; it is then possible to assume that the use of the QAM as the only single sign of sexual behavior is not efficient enough to detect cows in estrus in the warm tropics and supports what has recently been reported using rating tables considering other secondary signs as well [16], in accordance with the highest efficiency using a scale with seven signs, reported by [28]; and the possibility of more effectively predicting ovulation time by using a scale of points with the estrus signs [11, 14].

Some authors [23–25] have reported a low estrus detection rate for control cows subjected to AI (extreme values: 22.9% to 31.8%), which may be attributable to the traditional estrus detection system based VO of the QAM as the only sign of estrus; the small number and wide separation between two observations in a twelve-hour period; and the lack of trained personnel to recognize, to record, and to rank other secondary and important signs of this natural or induced physiological state, including the detection, recording, and tracking of sexual social events of formation of the active sexual groups, published by [3] for the DP cattle, which was reported as novel sign of estrus in specialized dairy herds [29]. System practices for the identification of the cow in estrus for crossbred populations that would indicate an inefficient estrus detection, which is identified as the most important factor limiting fertility of cattle in the world [9]. Also, it may be assumed that cows treated with PH-E and inseminated after being detected in estrus, and to which was applied between 1 mg and 1.5 mg of E-17*β* hormone 24 hours after removal of the intra-vaginal sponge. Hormone that act at the hypothalamus level and which is responsible for mounting behavior and estrus of the cow, which will be favoring the quiet mounting behavior considered in these papers, as the only sign of estrus. This hormone administrated in a dose of 500 *μ*g (0.5 mg) has been suggested as effective in inducing the behavioral estrus expression in cattle [30, 31]. Also, in high milk yield cattle, higher FSF rates have been reported while comparing VO and FTAI protocols [9, 32].

In DP livestock, however, results were 83.9% for estrus VO in contrast to 18.9% for FTAI, which were reported by [21]. Previous and promising reports with 38% of cows detected in spontaneous estrus without being observed in QAM with 57% of fertility strengthen and support the use of score table in order to identify cows to be assigned to AI [16].

In contrast, for tropical livestock, papers comparing FSF-FTAI and/or AI by detecting a single sign of estrus using rating tables were not found in any revision made, which may be attributable to a lack of interest among researchers, practitioners, and breeders of the knowledge of the sexual behavior of the cow and its value to improve reproductive efficiency and animal welfare on farms.

A significant association (*P* < 0.05) was detected between the FSF and the levels of DMY at the time of AI (Table 2), showing higher pregnant rates (46.15%) for cows with DMY ≥ 6 k/d and FSF of only 31.15% for cows with <6 k/d. These results contrast with the fact that an antagonistic relationship between milk yield and reproduction should be expected [33, 34]. On the other hand, this result could be attributable to the lengthening of lactation days.

The odds ratio for DMY for cows with less than 6 k/d compared to cows with DMY greater than 6 k/d was 0.5086 with a 95% CI (0.2664 to 0.9712). The meaning of this figure is that cows with DMY greater than 6 k/d had more chances to be pregnant; in fact, by using the equation given in Table 3, the odds ratio reverse can be found to be 1.9661, which indicates that high producing cows had 96.61% more chance of becoming pregnant at first service than those with lower DMY. This should be explained because of the management given to the cows, since the nutritional management given to these cows is better than the average found in most herds of the region.

## 4. Conclusions and Recommendations 

(1) Even when the SC-FTAI, protocol had higher pregnancy rates, it was not different from the NSE-AI protocol. Economic analysis should be performed in order to decide what AIP should be suggested for practical purposes. (2) The value of the OR for SC-FTAI was not significantly (*P* > 0.05) higher, so that the use of the NSE-AI protocol to inseminate the herd is not disposable and can help to improve the efficiency of the estrus detection VO technique to identify the cow to be assigned to AI and to improve reproductive efficiency of cattle reared in the warm tropics. (3) DMY was then the only effect that affected significantly FSF. (4) The remaining effects PPA, BCIA, and CN did not show statistical differences on FSF.

Further validation studies are recommended on the use of cow identification intended for AI using a scorecard of signs of estrus, adjusted for other environmental factors affecting FSF.

## Figures and Tables

**Table 1:** Sexual behavioral signs of cows in estrus.

Item	Main and secondary estrus signs	Score
1	Quiet acceptance (standing) to be mounted by teaser bull or cow (QAM)	100
2	Standing to disoriented mounts	60
3	Chin resting of bull or cow on rump	50
4	Alopecia by scraping or excoriation on tail head or rump	30
5	Vulva mucosa red or pink	20/5
6	Presence or discharge of cervical mucus	30
7	Supporting chin on rump of other cows	10
8	Frequent urination (more than three times)	10
9	Sniffing and licking the anogenital zone by another cow	10
10	Flehmen by bull or cow	10
11	Walking in circles with mutual sniffing of the genitalia	10
12	Butting head to head	5
13	Attempt or rejection to mount	5
14	To be followed by other cows (following)	5
15	Walking, agitated, or nervous (restlessness)	5
16	Accepting chin or head support else where	5

Score ≥ 100 points needed to classify a cow in estrus in an observation period AM or PM		

**Table 2:** *χ* ^2^ tests for factors associated with FSF in cows inseminated under two artificial insemination protocols (SC-FTAI and NSE-AI).

Factor levels	Diagnosis				Total		*χ* ^2^ test *P* value
	Pregnant		Open			
	*N*	%	*N*	%	*N*	%
SC-FTAI	46	42.20	63	57.80	109	100.00	χ^2^ = 0.0064 (*P* = 0.93)
NSE-AI	45	41.67	63	58.33	108	100.00	
Total (*N*)	**91**	**41.94**	**126**	**58.06**	**217**	**100.00**	

PPA ≤ 100 d	17	42.50	23	57.50	40	100.00	χ^2^ = 0.0064 (*P* = 0.93)
PPA > 100 d	74	41.81	103	58.19	177	100.00	
Total (*N*)	**91**	**41.94**	**126**	**58.06**	**217**	**100.00**	

DMY ≥ 6 k/L/d	72	46.15	84	53.85	156	100.00	χ^2^ = 4.05 (*P* = 0.04)
DMY < 6 k/L/d	19	31.15	42	68.85	61	100.00	
Total (*N*)	**91**	**41.94**	**126**	**58.06**	**217**	**100.00**	

BCAI ≥ 2.5	71	42.51	96	57.49	167	100.00	χ^2^ = 0.10 (*P* < 0.75)
BCAI < 2.5	20	40.00	30	60.00	50	100.00	
Total (*N*)	**91**	**41.94**	**126**	**58.06**	**217**	**100.00**	

CN ≤ 3	37	38.95	58	61.05	122	100.00	χ^2^ = 0.62
CN > 3	54	44.26	68	55.74	95	100.00	(*P* = 0.43)
Total (*N*)	**91**	**41.94**	**126**	**58.06**	**217**	**100.00**	

SC-FTAI: synchronized cows and fixed time artificial insemination, NSE-AI: natural estrus observation applying rating score table, PPA: postpartum anestrous, DMY: daily milk yield, BCAI: body condition at AI time, and CN = calving number.

**Table 3:** Odds ratios and confidence intervals for factors associated with FSF in cows under insemination based on fixed time or natural estrus observation applying a rating score table.

Predictor levels	Estimators		Probability of the likelihood ratio	Somers' D
	ODDS ratio	95% confidence interval		
SC-FTAI	1.050	0.595–1.855	0.4435	0.178
NSE-AI				
PPA ≤ 100 d	0.802	0.382–1.687		
PPA > 100 d				
DMY ≥ 6 k/L/d	0.509	0.266–0.971		
DMY < 6 k/L/d				
BCAI ≥ 2.5	0.850	0.440–1.642		
BCAI < 2.5				
CN ≤ 3	0.832	0.479–1.445		
CN > 3				

Logistic prediction equation:				
$\text{Prob}(y=1|\text{predictors)}=\frac{e^{0.1435+0.0491\text{AIP}-0.2203\text{AAP}-0.6761\text{DMY}-0.1627\text{BCAI}-0.1840\text{PN}}}{\left(1+e^{0.1435+0.0491\text{AIP}-0.2203\text{AAP}-0.6761\text{DMY}-0.1627\text{BCAI}-0.1840\text{PN}}\right)}$				

AIP = protocol type: SC-FTAI: synchronized cows and fixed time artificial insemination; NSE-AI: natural estrus observation applying rating score table; PPA: postpartum anestrous; DMY: daily milk yield; BCAI: body condition at AI time; CN: calving number.
